# Infant Milk Feeding Influences Adult Bone Health: A Prospective Study from Birth to 32 Years

**DOI:** 10.1371/journal.pone.0019068

**Published:** 2011-04-27

**Authors:** Satu Pirilä, Mervi Taskinen, Heli Viljakainen, Merja Kajosaari, Maila Turanlahti, Ulla M. Saarinen-Pihkala, Outi Mäkitie

**Affiliations:** Children's Hospital, Helsinki University Central Hospital and University of Helsinki, Helsinki, Finland; Brigham and Women's Hospital, and Harvard Medical School, United States of America

## Abstract

**Background:**

Peak bone mass, attained by early adulthood, is influenced by genetic and life-style factors. Early infant feeding and duration of breastfeeding in particular, associate with several health-related parameters in childhood. The aim of this study was to examine whether the effects of early infant feeding extend to peak bone mass and other bone health characteristics at adult age.

**Methods and Findings:**

A cohort of 158 adults (76 males) born in Helsinki, Finland, 1975, prospectively followed up from birth, underwent physical examination and bone densitometry to study bone area, bone mineral content (BMC), and bone mineral density (BMD) at 32 years of age. Life-style factors relevant for bone health were recorded. For data analysis the cohort was divided into three equal-size groups according to the total duration of breastfeeding (BF): Short (≤3 months), Intermediate and Prolonged (≥7 months) BF groups. In males short BF is associated with higher bone area, BMC, and BMD compared to longer BF. Males in the Short BF group had on average 4.7% higher whole body BMD than males in the Prolonged BF group. In multivariate analysis, after controlling for multiple confounding factors, the influence of BF duration on adult bone characteristics persisted in males. Differences between the three feeding groups were observed in lumbar spine bone area and BMC, and whole body BMD (MANCOVA; p = 0.025, p = 0.013, and p = 0.048, respectively), favoring the Short BF group. In women no differences were observed.

**Conclusions:**

In men, early infant milk feeding may have a significant impact on adult bone health. A potential explanation is that the calcium and phosphate contents were strikingly higher in formula milk and commercial cow milk/cow milk dilutions as opposed to human milk. Our novel finding merits further studies to determine means to ensure optimal bone mass development in infants with prolonged breastfeeding.

## Introduction

Several epidemiological studies have suggested that environmental factors during the sensitive phases of development, especially the fetal period and infancy, modify organ function and may have life-long consequences on an individual's risk of several common adult diseases, including ischemic heart disease, type 2 diabetes, obesity, and osteoporosis [1]–[3]. Nutrition during infancy, while contributing to growth and over-all health during childhood, may thus have a significant impact on various adult health-related parameters.

Peak bone mass, attained by early adulthood, is a major determinant of fracture risk and osteoporosis at adult age [4]. Up to 80% of peak bone mass is determined by genetic factors [5]. However, life-style factors, including calcium and vitamin D intake, as well as hormonal factors also play important roles in bone mass accrual, bone growth, and bone strength. There is increasing evidence that prenatal factors and nutrition in early infancy have long-term effects on the skeleton but it is not known whether these influences persist until adult age [6]–[8].

During the first few months of life the baby's essential nutrition is milk, either breast milk or a milk formula. Breastfeeding (BF) is known to have several beneficial effects on health during childhood, and some of these may extend into adulthood [9], [10]. BF protects against infections [11]–[15] and allergies [16], [17], and modifies growth and body composition [6], [18], [19]. It is not known whether these early nutritional differences translate into bone mass accrual or magnitude of peak bone mass. Only few studies have examined the long-term effects of BF on bone health. The findings have been controversial, some studies showing BF to have beneficial effects on bone mass until age of 8 years [7] while others have reported no effect [20] or a negative effect [6] on bone mass during early childhood. Earlier studies assessing the impact of BF on bone health at adult age the data on duration of BF were collected retrospectively [21]; to our knowledge the prospective studies have not extended until the age of peak bone mass attainment.

We have carried out a prospective cohort study from birth until 32 years of age to assess long-term health effects of BF. In this cohort details of early milk feeding were prospectively collected and well documented from the beginning of the study [19], [22], [23]. The purpose of the present study was to examine the impact of BF on peak bone mass and other bone health characteristics at adult age.

## Methods

This prospective, single center study with extended follow-up evaluated the impact of early infant feeding on adult health. Study protocol was approved by the Institutional Review Board of the Helsinki University Central Hospital. A written informed consent was obtained from all study subjects.

### Original cohort

The original cohort comprised 238 healthy, full-term Finnish infants with a birth weight exceeding 3000 g, born at the Helsinki University Central Hospital during the first three months of 1975. The initial purpose was to examine the role of milk feeding in iron status during infancy [22]–[25]. The mothers were encouraged to breastfeed for as long as possible. When breast milk became insufficient, instructions were given to use an adapted baby formula (Bona®, Chymos Oy, Finland) or a home-prepared cow's milk formula. Solid foods were introduced to all babies at 3.5 months according to the same strict instructions. All babies were examined by one of the authors (UM S-P) seven times during their first year: at 2 weeks and at 1, 2, 4, 6, 9, and 12 months of age. At each visit, data on feeding and anthropometric measurements were collected and venous blood drawn for laboratory tests. During the time of the initial study, the local official recommendation was to supplement all infants with 1000 IU (25 µg) of vitamin D, which was followed. Of the original cohort approximately 160 were also examined at 3, 5, 10 and 17 years of age by one of the authors (MK) [16], [17].

Information on BF and other milk feeding was prospectively collected at each visit until the 12 month visit. For the present study the subjects were divided into three equal size groups based on the total duration of BF. Consequently, the cut-off points for short and prolonged BF were defined as 3 months and 7 months. The results are presented separately for the three groups: 1. Short BF (≤3 mo), 2. Intermediate BF (>3 but <7 months), and 3. Prolonged BF (≥7 mo).

### Study cohort

All 238 subjects of the original cohort were traced through the Population Registry Centre in Finland. During 2006 to 2008 we invited them to participate in a study evaluating adult health. Altogether 188 subjects were reached and 158 (84% of those who responded; 66% of the whole cohort) consented to participate and were examined at the outpatient clinic at the Hospital for Children and Adolescents, Helsinki University Central Hospital, Finland. Those 30 who did not consent were distributed equally within the three feeding groups. Males were over-represented (60% males) but no other differences were observed between the non-participants and participants. The reasons for non-participation were lack of time or lack of interest. All participants were interviewed and underwent a physical examination by one of the authors (SP) who was blinded to the previous data and the feeding history.

### Clinical history

All participants filled in a structured questionnaire on present health, socio-economic background, pregnancies, medications, diet, consumption of dairy products, use of vitamin supplements, physical activity, fracture history, use of alcohol, and smoking; the information was completed by interview at the study visit. A three-day food record was collected to assess nutrient intakes including calcium and vitamin D; it was completed by 147 participants (93%), and analyzed by a licensed dietician (software AIVO 2000 - Diet32, Version 1.4.6.2). In the 11 subjects who did not return their food record, the intake of calcium was estimated by the consumption of dairy products, as this correlated with the intake of calcium from food records in the rest of the sample (r = 0.44, p<0.001). Leisure time physical activity was graded from 1 to 5 according to frequency, duration, and intensity of exercise, 1 representing no leisure time exercise and 5 representing hard training more than twice a week. Physical activity was graded separately for teenage years and for the current time. Teenage exercise data were not available for 6 participants; missing values were replaced with cohort mean values in multivariate analysis.

### Anthropometric measurements

Weight and height were measured in light clothing and without shoes. Weight to the closest 0.1 kg and height to the closest 0.1 cm were recorded. An electric scale was used for weight, and a standard measuring column for height. These height and weight measures were used to calculate body mass index (BMI kg/m^2^).

### Biochemical studies

Venous blood was drawn at 9 am after an overnight fast for serum/plasma biochemistry. The second morning void, with a 2-hour interval from the first void, was used for urine biochemistry. Serum bone-specific alkaline phosphatase (BALP) was assessed by agarose gel electrophoresis, vitamin D (25-OHD) by HPLC [26], osteocalcin by electrochemiluminescence immunoassay (The Roche Elecsys 1010/2010,Indianapolis, IN, USA), carboxy-terminal telopeptide of type I collagen (ICTP) and procollagen type I N-terminal propeptide (PINP) by radioimmunoassay (Orion Diagnostica Oy, Espoo, Finland). The urine sample was analyzed for N-terminal telopeptide (NTX) by enzyme linked immunosorbent assay (Wampole Laboratories, Princeton, NJ, USA).

### Bone densitometry

All subjects underwent bone mineral density (BMD) measurement with dual energy x-ray absorptiometry (DXA). Bone mineral content (BMC), BMD, and bone area for the lumbar spine (LS) (L1-L4), femoral neck (FN) and whole body (WB) were measured with DXA (Hologic Discovery A, software version 12.4∶3, Waltham, MA, USA). The BMDs were transformed into Z-scores by using age- and sex-specific reference data for the equipment. In order to identify possible vertebral compression fractures an antero-posterior and lateral image of the thoracic and lumbar spine (Instant Vertebral Assessment, IVA) was also obtained with DXA. Compressions were assessed according to the classification by Genant et al [27].

### Statistical analyses

Statistical analyses were performed with SPSS software (version 17.0.1.). One-way ANOVA was used to compare background characteristics in the three study groups and to compare bone variables before adjusting for confounding factors. Chi-square test was used to assess relationships between categorical independent variables. Pearson correlation was used to identify confounders for the bone variables. The potential confounding factors tested were gender, dietary intake of calcium, teen-age and current physical activity, serum 25-OHD concentration, smoking history, alcohol consumption, pregnancies, fractures, weight, height, BMI, weight changes during adult life, and birth weight. In linear regression, a backward method was applied to prioritize the confounding factors. Subsequently, the identified main confounders were gender, weight, teen-age physical activity, dietary intake of calcium, BMI and alcohol consumption. Partial correlation was used to build up the model for multivariate analysis. In linear regression and partial correlation the duration of BF was used as a continuous variable. Multivariate analysis of covariance (MANCOVA) was applied to test whether the early life feeding pattern affected bone health in adulthood after controlling for confounding factors. In the whole cohort we used LS, FN and WB BMD Z-scores to allow comparison between the genders. Because of a significant interaction between feeding group and gender, we stratified the cohort according to gender. After stratification crude BMD, BMC, and bone area values were used.

Similarly, we identified gender, current physical activity, alcohol consumption, calcium intake, and BMI as significant confounders for bone turnover markers. Logarithmic transformations were used in MANCOVA to satisfy the assumption of normality. Dependent variables were BALP, PINP, ICTP, Osteocalcin, and NTX.

Preliminary assumption testing was conducted before multivariate analyses to check for normality, linearity, univariate and multivariate outliers, and for homogeneity of variance matrices, and no serious violations were noted.

## Results

### Cohort characteristics

Altogether 158 subjects, 76 males and 82 females (median age 32.6 years, range 31.7–34.0 years) participated in this follow-up study. They represented 66% of the original cohort (73% of the women and 60% of the men). The numbers of participants in the three groups were 56, 48 and 54 for Short, Intermediate, and Prolonged BF, respectively (Table 1). There were no differences in birth weight or current weight, height or BMI between the feeding groups (Table 1).

**Table 1:** Cohort characteristics.

	Short BF n = 56	Intermediate BF n = 48	Prolonged BF n = 54	Total n = 158	P
Age (years) (range)	32.6 (31.7–33.7)	32.8 (31.8–34.0)	32.5 (31.7–33.6)	32.6 (31.7–34.0)	0.07
Males %	46%	44%	54%	48%	0.58¶
Males (n)	26	21	29	76	
Weight (kg)^1^	85.3 (±13.6)	83.2 (±9.6)	82.5 (±12.5)	83.7 (±12.1)	0.68
Height (cm)^1^	181.8 (±7.6)	179.8 (±5.5)	179.2 (±6.1)	180.3 (±6.5)	0.33
BMI (kg/cm^2^)^1^	25.8 (±3.6)	25.7 (±2.6)	25.6 (±3.5)	25.7 (±3.2)	0.98
Birth weight (kg)^1^	3.74 (±0.35)	3.65 (±0.40)	3.72 (±0.42)	3.70 (±0.39)	0.72
Females (n)	30	27	25	82	
Weight (kg)^1^	67.8 (±9.6)	65.4 (±12.7)	68.4 (±11.2)	67.2 (±11.1)	0.59
Height (cm)^1^	167.7 (±6.3)	165.7 (±5.1)	169.1 (±6.6)	167.4 (±6.1)	0.13
BMI (kg/cm^2^)^1^	24.2 (±3.7)	23.9 (±4·7)	23.9 (±3.8)	24.0 (±4.0)	0.96
Birth weight (kg)^1^	3.56 (±0.35)	3.47 (±0.33)	3.57 (±0.30)	3.53 (±0.33)	0.44
Pregnancies per	2.1	2.1	1.7	1.9	0.54
women (mean)^2^					
No pregnancies	50%	59%	32%	48%	0.14¶
Calcium intake (mg/day)^1^	1360 (±560)	1100 (±390)	1170 (±430)	1220 (±480)	0.014
Vitamin D intake (µg/day)^1^	7.6 (±5.2)	6.8 (±4.8)	8.4 (±5.5)	7.7 (±5.3)	0.33
Serum 25 (OH)D (nmol/L)^1^	46.7 (±15.6)	50.5 (±16.9)	44.3 (±12.3)	47.0 (±15.1)	0.11
Physical activity^4^					
At 10 to 20 years	3.7	3.6	3.5	3.6	0.56
Current	3.2	3.2	3.2	3.2	0.87
Smokers (n)	17	7	13	37	
%	30%	15%	24%	23%	0.17¶
cigarettes/day^3^	14	9	14	13	0.31
Fractures					
None	52%	58%	52%	54%	0.75
Level of education, high school or more	77%	81%	76%	78%	0.79
Alcohol usage, ≥ 3 times a week	11%	17%	24%	24%	0.67¶

P values were calculated with ANOVA or with chi-square test (marked with ¶).

1 Mean values and standard deviations.

2 Mean numbers of pregnancies in women with at least one pregnancy.

3 Mean amounts of cigarettes among those who smoke.

4 Physical activity scaled from 1 to 5; 1 representing no exercise and 5 representing hard training more that twice a week.

The cohort lifestyle characteristics are shown in Table 1. Most of the participants (87%) regarded their own health good or excellent. Only 15% used a prescribed medication regularly. The rate of physical activity was high: 43% exercised regularly, 29% reported light daily physical exercise, 25% exercised occasionally, and 2% were not physically active. Regarding exercise during the teen-age years (at 10–20 years of age), 60% reported exercise more than twice a week. There were no differences between the feeding groups in these lifestyle characteristics. The socio-economic background was also similar in all feeding groups. The average level of education in each group was higher than in the general population; 78% of the subjects were at least high-school graduates.

Based on the three-day food records, calcium intake was adequate (at least 800 mg daily) in 82% of the participants. Dairy products were the main source of calcium in all subjects and the total intake of calcium strongly associated with dairy consumption (r = 0.44, p<0.001); no differences were observed between the feeding groups. The mean daily intake of calcium was higher in the Short BF group than in the other two groups (ANOVA; p = 0.014). In men, the intake of calcium differed significantly between the feeding groups (ANOVA; p = 0.001), while no difference was observed in women (p = 0.33). Daily intake of calcium was below 800 mg in 9%, 25%, and 20% in groups with Short, Intermediate, and Prolonged BF, respectively (Chi-square; p = 0.083). Intake of vitamin D was similar in all feeding groups, but in only 40% the intake was in accordance with the recommendation (≥7.5 µg per day).

Most participants (87%) used alcohol up to twice a week, and 13% more than 3 times a week. Twenty-four percent of the cohort smoked regularly, and 21% were former smokers.

### Skeletal findings

In the whole cohort no differences were observed in BMD Z-scores in any skeletal sites between feeding groups tested with ANOVA. In the multivariate analysis using gender, weight, dietary intake of calcium, and physical activity at adolescence as covariates, no difference in LS, FN, and WB were observed between the feeding groups (MANCOVA; p = 0.29, p = 0.41, and p = 0.29, respectively) (Table 2). However, an interaction was marked with gender (MANCOVA; p = 0.001, p = 0.10, and p = 0.011 in LS, FN, and WB BMD Z-scores, respectively) and therefore the effect of feeding group on BMD, BMC, and bone area was studied separately in males and females.

**Table 2:** BMD Z-scores and bone areas in the three study groups.

			Short BF		Intermediate BF		Prolonged BF		P
**Whole group**			**n = 56**	**SD**	**n = 48**	**SD**	**n = 53**	**SD**	
	Z-score	LS	−0.06	1.03	−0.20	1.02	−0.38	1.02	0.29
		FN	−0.01	0.89	−0.09	0.89	−0.24	0.88	0.41
		WB	0.29	0.94	0.21	0.93	0.02	0.93	0.29
**Males**			**n = 26**		**n = 21**		**n = 29**		
	LS	Z-score	−0.08	1.00	−0.44	0.98	−0.67	1.00	0.12
		Bone area (cm^2^)	72.0	5.4	69.1	5.2	67.8	5.2	0.025
	FN	Z-score	0.07	0.89	0.03	0.84	−0.38	0.85	0.10
		Bone area (cm^2^)	5.9	0.5	5.8	0.4	5.8	0.4	0.74
	WB	Z-score	0.22	0.95	0.08	0.89	−0.35	0.91	0.049
		Bone area (cm^2^)	2354.4	127.6	2364.3	120.7	2314.7	122.3	0.30
**Females**			**n = 30**		**n = 27**		**n = 24**		
	LS	Z-score	0.08	1.00	0.06	1.01	−0.27	1.00	0.37
		Bone area (cm^2^)	59.5	326.7	60.0	311.5	60.6	297.0	0.81
	FN	Z-score	−0.04	0.91	−0.14	0.92	−0.17	0.92	0.86
		Bone area (cm^2^)	5.1	28.0	5.0	25.8	5.1	24.9	0.27
	WB	Z-score	0.43	0.96	0.41	0.97	0.26	0.97	0.78
		Bone area (cm^2^)	2017.7	124.7	1998.1	126.8	2040.3	126.5	0.48

P values were analyzed with MANCOVA. For Z-scores the covariates used were gender (in the whole group), current weight, calcium intake, and physical activity at adolescence. For bone area the covariates were calcium intake, current weight and physical activity at adolescence. LS = lumbar spine; FN = femoral neck; WB  =  whole body; SD  =  standard deviation.

**Figure 1:**
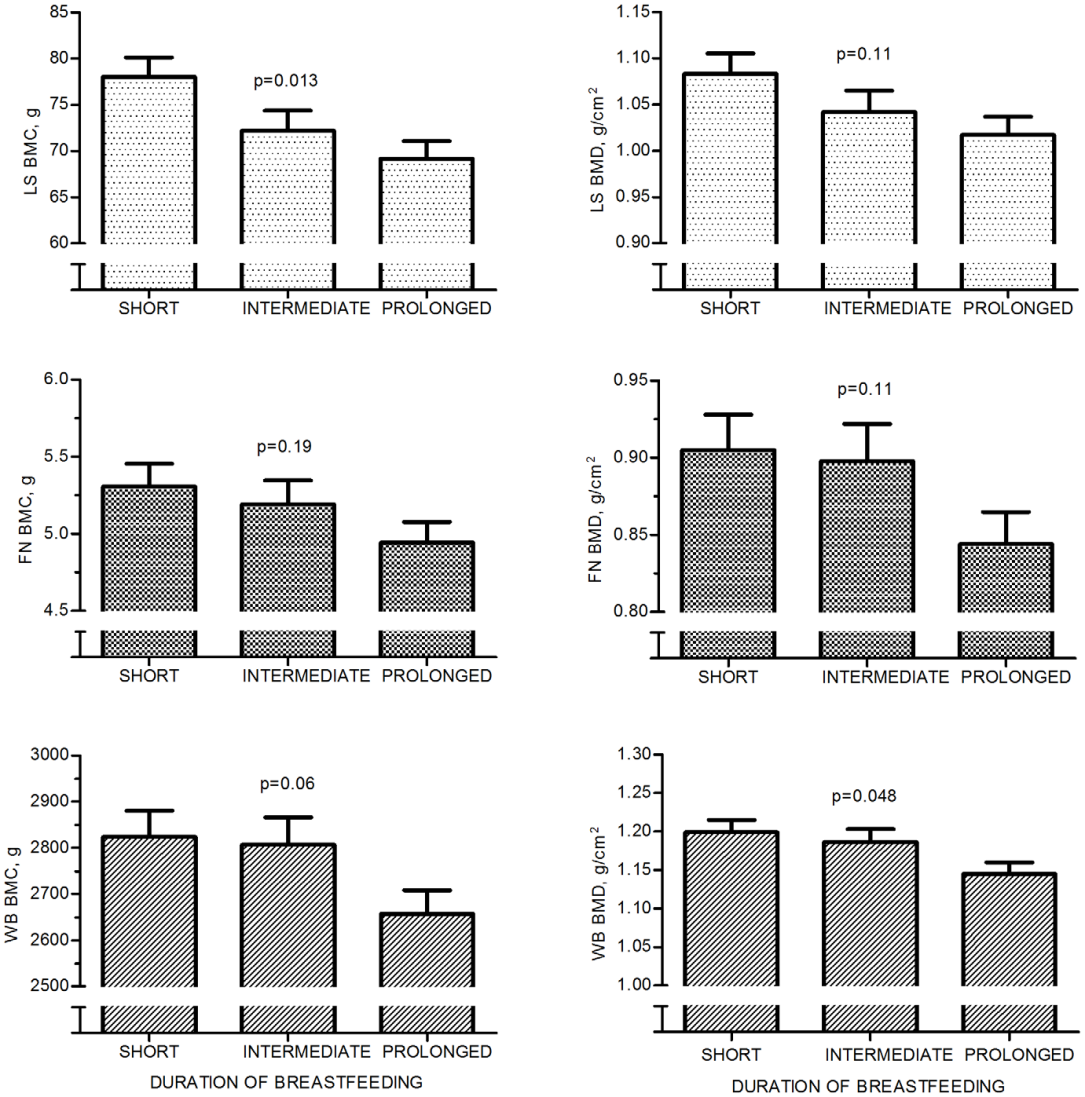
BMD and BMC in the different feeding groups in males. Covariates used were current weight, daily intake of calcium and physical activity at adolescence. BMD  =  bone mineral density; BMC  =  bone mineral content; BF  =  breast feeding (Short ≤3 mo; Intermediate >3 mo, but <7 mo; Prolonged ≥7 mo); LS  =  lumbar spine; FN  =  femoral neck; WB  =  whole body.

In males there were differences between the feeding groups, even before adjusting for confounding factors, in LS BMC (ANOVA; p = 0.026) and LS bone area (ANOVA; p = 0.003); the Short BF group showing the highest values. However, the effect may be due to confounding factors as the current dietary intake of calcium was highest in Short BF group. In multivariate analysis, after controlling for the confounding factors (dietary intake of calcium, current weight and physical activity at adolescence), the duration of BF showed an influence on bone characteristics in males. Significant differences between the feeding groups were observed in LS BMC, LS bone area, and WB BMD (MANCOVA; p = 0.013, p = 0.025, and p = 0.048, respectively) (Table 2, Figure 1). A similar trend was also observed in WB BMC, again in favor of the short BF group (MANCOVA; p = 0.061) (Figure 1). In pairwise comparison the Short BF group had higher LS BMC, LS bone area, and WB BMD when compared with the Prolonged BF group (pairwise comparisons with Bonferroni adjustment; p = 0.010, p = 0.022, and p = 0.066, respectively). The difference in WB BMD between the Short and Prolonged BF group was 4.7% favoring early weaning. In contrast, in females there were no differences between the feeding groups in any skeletal parameters before or after adjusting for confounding factors.

Forty-six percent of the subjects had a history of at least one peripheral fracture; 29% of the subjects had fractured before age 18 years. Minor fractures (digits, ribs or nose) were common and only 39% of all fractures were considered significant (forearm, upper arm, lower leg or upper leg fractures). Seven fractures resulted from high energy trauma and none were considered osteoporotic. Skeletal findings did not differ between the subjects with and without fractures. No differences in fracture prevalence were observed between the feeding groups (Chi-square; P = 0.75). Review of the spine images, obtained by DXA, revealed no compression fractures in any of the subjects.

The biochemical markers of bone turnover were analyzed in multivariate analysis. The covariates used were gender, current physical activity, and BMI. There were no differences between the BF groups in any of the measured bone turnover parameters (Table 3).

**Table 3:** Multivariate analysis of biochemical bone markers between the feeding groups.

	Normal range	Short BF	SEM	Intermediate BF	SEM	Prolonged BF	SEM	P
**Whole group**		**n = 34**		**n = 36**		**n = 36**		
BALP (U/L)		29.4	1.8	29.4	2.0	26.1	2.0	0.38
Osteocalcin (µ/L)	4.9–30.9 (F) 3.2–39.6 (M)	20.5	1.3	20.0	1.4	19.50	1.4	0.91
PINP (µ/L)	19–84	49.1	2.4	48.6	2.6	46.1	2.6	0.85
ICTP (µ/L)	1.5–5	3.7	0.2	3.4	0.3	3.3	0.3	0.53
NTX (nmol/mmol)	<65	46.9	4.3	48.7	4.7	50.7	4.7	0.97
**Males**		**n = 22**		**n = 16**		**n = 19**		
BALP (U/L)		32.9	2.5	32.6	2.7	27.6	2.6	0.29
Osteocalcin (µ/L)	3.2–39.6	22.0	2.0	20.2	2.2	17.2	2.1	0.55
PINP (µ/L)	19–84	51.4	3.2	51.3	3.6	44.1	3.7	0.29
ICTP (µ/L)	1.5–5	3.9	0.4	3.2	0.5	3.0	0.5	0.32
NTX (nmol/mmol)	<65	46.6	7.8	54.3	8.6	56.2	8.1	0.89
**Females**		**n = 21**		**n = 20**		**n = 17**		
BALP (U/L)		24.8	2.9	26.6	2.9	25.8	3.2	0.91
Osteocalcin (µ/L)	4.9–30.9	20.2	1.8	19.5	1.8	21.0	2.0	0.87
PINP (µ/L)	19–84	47.5	3.9	46.1	4.0	47.9	4.4	0.95
ICTP (µ/L)	1.5–5	3.7	0.2	3.6	0.2	3.7	0.2	0.93
NTX (nmol/mmol)	<65	42.9	4.7	44.6	4.7	49.2	5.2	0.67

The covariates used were for the whole group BMI, current physical activity, and gender; for males BMI, calcium intake and current physical activity; for females BMI, current physical activity, and alcohol consumption. BALP  =  bone specific alkaline phosphatase; PINP  =  procollagen type I N-terminal propeptide; ICTP  =  C-terminal telopeptide of type I collagen; NTX  =  N-telopeptide; SEM  =  standard error of mean.

## Discussion

To our best knowledge this is the first study to assess long-term skeletal consequences of early infant feeding prospectively from infancy to young adulthood. Several studies have shown that duration of breastfeeding influences bone mass beyond infancy [7], [8] but the persistence, direction, and magnitude of this effect have remained unclear. Our results suggest that early infant feeding may have long-term effects on skeletal health.

Our main finding was that males who were breastfed for less than 3 months had at 32 years of age higher lumbar spine BMC and bone area, and higher whole body BMC and BMD than males with prolonged breastfeeding. In whole body BMD the difference between short and prolonged BF groups was 4.7% after adjusting for confounders. This is a clinically significant finding. However, the duration of breastfeeding had no impact on skeletal parameters in females at the age of 32 years. These results were unexpected and somewhat surprising.

Previous findings regarding the impact of BF on bone health have been conflicting. Jones et al. reported effects of BF on bone mass at 8 years; children breastfed for more than 3 months had higher LS, FN, and WB BMD than those breastfed for less than 3 months; the effect was same in both genders [7]. In the Southampton prospective study, 318 boys and 281 girls were examined at 4 years; the duration of breastfeeding was not related to BMD or bone area in either boys or girls [20]. In the Newcastle study with a nearly 50 years' follow-up, the early life factors played a greater role for bone health in males than in females, in agreement with our findings. They showed that infant feeding influenced adult skeletal strength indirectly by affecting the achieved adult body size [21].

Our study cohort was carefully assessed for lifestyle factors known to influence bone health, such as smoking, physical activity, illnesses and medications, diet, alcohol consumption, and pregnancies in women. Dietary intake of calcium and physical activity are important determinants of BMD [28]. Men in the Short BF group had slightly higher mean dietary intake of calcium than men in the other groups. However, in all groups the mean intake of calcium exceeded the current recommendation (800 mg/d) (Nordic Nutrition Council, 2004) [29]. Dairy products, and not supplements, were the main source of calcium in all subjects, which is typical for the Finnish diet. Current physical activity and especially physical activity during adolescence correlated with adult BMD and BMC. However, the differences between the feeding groups in males persisted even after adjusting for current weight, intake of calcium, and physical activity in adolescence, suggesting that in males, early milk feeding plays a major role in bone mass accrual and influences the magnitude of peak bone mass.

In females no differences between the three feeding groups were observed in any bone-related parameters. It seems that in females, the current lifestyle and hormonal factors play a greater role in bone health than infant feeding. We collected data on pregnancies, use of contraceptives and duration of breastfeeding of their own children, but were unable to demonstrate a conclusive role for any of these as the major bone health determinant (data not shown). Pregnancies and lactation are shown to have deleterious effects on the mother's skeleton [30]. Similarly, oral contraceptives may influence bone mass [31]. It is therefore not surprising that these more recent life-style factors probably superseded the effects of feeding patterns in infancy.

An explanation for our main finding is offered by the constitutions of the milks consumed during the first year of life (Table 4). In 1975, when the participants first were enrolled in this prospective study, the main focus was at the protein content, especially whey and casein, of the milk. Cow milk diluted 1∶1 with water, with added sugar, was also commonly used at that time. In our cohort the babies younger than 6 months used a 3∶2 dilution of cow milk and water, with added lactose 50 g per liter, according to careful instructions. Babies over 6 months of age used commercial cow milk as such. There are quite striking differences in calcium and phosphorus contents of these milks. The calcium content was 1.4 times higher in the formula, 3.1 times higher in the cow milk dilution, and 4.4 times higher in the commercial cow milk, as compared with breast milk. The phosphorus content was 2.6, 6.3, and 12 times higher, respectively, when compared with breast milk. Also protein content is on average 20% higher in diluted milk and in milk formula compared to breast milk (Table 4). Since milk is the most important source of nutrition during the first year of life - the period when the individual triples his/her weight, and is also in a particularly sensitive phase of development - such striking differences in calcium, phosphorus and protein intake are likely to be of major importance for skeletal growth and structure. For the Short BF group calcium, phosphorus and protein rich infant formula or cow's milk dilution was introduced earlier than for the other groups. Unfortunately no bone densitometry measurements were performed to our cohort during childhood.

**Table 4:** Components of human milk, infant formula, and cow milk dilution used in the study Nutritive values per 100 g.

	Human milk^1^	Infant formula^2^ (Bona®, Chymos)	Cow milk dilution^3^ <6 months	Cow milk^4^ >6 months
Energy kJ (kcal)	303 (72)	283 (68)	184 (44)	220 (53)
Protein total (g)	1.5	1.5	2.1	3.0
Carbohydrate (g)	6.5	7.2	3.3	4.8
Fat total (g)	4.5	3.5	2.6	2.5
Minerals				
Calcium (mg)	27.0	38.0	86.1	120.0
Phosphorus (mg)	10.0	26.0	62.5	90.0
Iron (mg)	<0.1	0.5	<0.1	<0.1
Sodium (mg)	20.0	15.0	30.5	41
Vitamins				
Vitamin D (µg)	<0.1	0	0	0

1 National Institute for Health and Welfare, Nutrition Unit, Finland, Based on the Fineli Food Composition Database Release 10 (June 30, 2009), http://www.fineli.fi/food.php?foodid=603&lang=en.

2 Chymos, Finland, Bona Infant Formula carton 1974.

3 Home-made cow milk dilution: for infants below 6 months of age: 600 ml of dairy milk (3.9%fat) and 400 ml of water mixed and brought to the boiling point, and 50g lactose added.

4 Infants over 6 months: commercially available low fat (2.5%) milk used as such.

A recent study on piglets showed that those fed with cow milk formula or soy milk had higher BMD and BMC values than breastfed piglets. Further, the cow milk and soy milk fed piglets had higher osteoblast and lower osteoclast numbers, and higher serum bone formation markers than the BF piglets [32]. This observation was speculated to signify that early childhood nutrition directs stem cell differentiation and programs skeletal development. Similar to these observations we found the highest bone mass-related parameters in subjects who were exposed to formula early. However, no differences in bone turnover markers were observed in our cohort.

Intestinal calcium absorption is dependent on serum vitamin D concentration. In 1974 and 1975, when the mothers of our study subjects were pregnant, the need for sufficient calcium and vitamin D were not emphasized during pregnancy. We have no data on dietary habits of these pregnant mothers. All our study subjects were born during winter months, i.e., between January and March when the serum levels of vitamin D are known to be lowest. All babies were substituted with 1000 IU vitamin D daily, according to the official recommendation in 1975. Since the 1990′s the recommendation has been decreased to 400 IU. We recently showed that today nearly 70% of pregnant women are vitamin D deficient, and that maternal vitamin D status influences bone characteristics of the newborn baby [33]. It is possible that a significant proportion of the mothers in the present study cohort were vitamin D deficient, which may have life-long effects on bone health in the offspring [34].

Many potential benefits of breastfeeding are well-known, including e.g. prevention of infections [11], [13], atopic disease [16], [17], and iron status [23]–[25], as well as many psycho-social and developmental aspects [35]. That prolonged breastfeeding seems to be suboptimal for skeletal health is a novel finding. The infant's inadequate intake of calcium, phosphate and protein during breastfeeding may need to be considered especially with prolonged breastfeeding.

Our study has major strengths but also some limitations. The main strength is that the dietary data were carefully prospectively collected since birth. All subjects visited the outpatient clinic seven times during their first year of life and the total duration of breastfeeding was carefully documented. They were also seen during childhood and teen-age years [16], [17] but data on nutrition and other life-style factors were not prospectively collected during follow-up and retrospective collection of data e.g. on nutrition was considered unreliable. However, longitudinal changes in some life-style factors, such as physical activity, smoking habits, major weight gains or losses and pregnancies, were taken into account. Fractures were not radiologically confirmed; this may have resulted in the overall high rate of reported fractures. The fracture rate during childhood was comparable to recent epidemiological data [36] and most of the fractures were considered minor. Unfortunately we did not have earlier bone mass measurements for the subjects and it thus remains unknown when the differences between the feeding groups appeared and when they were most pronounced. However, since peak bone mass is an important predictor for life-time risk of osteoporosis the observed differences at young adult age may be more important than possible changes observed earlier. DXA has some limitations in bone health assessment and optimally other measurement techniques, i.e. pQCT, could have provided more detailed information about the differences in bone health characteristics.

In conclusion, our extended follow-up study from birth to 32 years suggests that early infant feeding has an influence on bone mass in males, extending beyond the age of peak bone mass attainment. Compared with prolonged breastfeeding, early formula feeding associated with higher adult bone mass in males. This is a novel finding. One important difference in early feeding was the intake of calcium, phosphate, and protein, the concentrations being high in formula milk and cow milk dilutions, but strikingly lower in human milk. While breastfeeding has many benefits for the baby, prolonged breastfeeding might jeopardize optimal bone mass attainment. The role of infant feeding, particularly calcium and phosphate intake, in skeletal health merits further studies.
